# Mapping functional connectivity of bursting neuronal networks

**DOI:** 10.1007/s41109-017-0037-0

**Published:** 2017-06-19

**Authors:** Tuan D. Nguyen, Kelly D. O’Connor, Krishna Sheth, Nick Bolle

**Affiliations:** The College of New Jersey, Department of Physics, Ewing, 08628 NJ USA

**Keywords:** Calcium imaging, Laser scanning photostimulation, Neuronal network, Network bursting

## Abstract

Using single-cell laser scanning photostimulation (LSPS) combined with broad-field calcium imaging, we measured the functional connectivity of neuronal cultures before and after the developmental appearance of network bursting. From these data, network properties were determined for these relatively large neuronal networks. Based on these properties, we found that although ‘small-world’ network behavior existed throughout this time period, only average node degree and global efficiency correlate with the development of network bursting while clustering and local efficiency remained relatively constant.

## Introduction

Coherent, large-scale activity of neurons, known as neuronal oscillations, is an emergent phenomenon found in many neural systems. In the intact brain, neuronal oscillations have been linked to sleeping states, memory formation, perception, and motor control (Buzsáki and Draguhn 2004; Fell and Axmacher 2011). Outside the brain, they have been observed in both acute brain slices (Kubota et al. 2003) and neuronal cultures, where they are more commonly known as network bursts (Segev et al. 2001; Wagenaar et al. 2006). It has been suggested that, within all of these systems, neuronal oscillations play the important role of facilitating long-distance communication between large populations of neurons (Fries 2005).

A great challenge has been understanding what role the underlying network structure plays in producing synchronization (Fuchs et al. 2009; Maheswaranathan et al. 2012). Knowledge of connectivity has been difficult to obtain even for small neuronal cultures consisting of ∼10^3^ neurons and ∼10^5^ connections. As a result, much effort has been focused on the converse problem of deducing connectivity from analyses of network activity (Jia et al. 2004; Tibau et al. 2013; Timme 2007; Savarraj and Chiu 2014).

In this article, we present direct measurements of functional connectivity using a technique that performs laser scanning photostimulation (LSPS) of single neurons with simultaneous calcium (Ca) imaging of a large cell-population. To our knowledge, such a combination of methods has not been done before. This technique allows rapid functional mapping of excitatory connections in neuronal networks consisting of 150-200 neurons and 1500-2000 connections. Throughout a 12-day period, connectivity maps were made, from which network properties before and after the beginning of network bursting were determined.

## Apparatus and materials

**Fig. 1:**
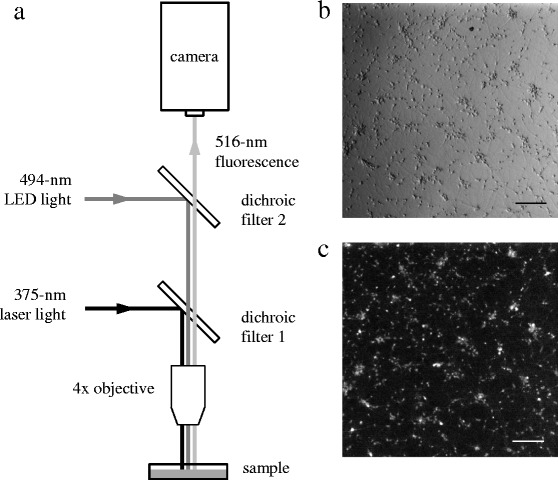
**a** Schematic diagram of the apparatus. Field-of-view (FOV) **b** under Bright-field illumination and **c** of corresponding calcium indicator (Fluo-4) fluorescence. Scale bars: 200 *μ*m

Both LSPS (Katz and Dalva 1994; Sturm et al. 2014) and Ca imaging (Cossart et al. 2005; Grienberger and Konnerth 2012) have been used successfully to respectively stimulate and record activity from a large population of neurons. We combined these techniques by modifying an existing microscope (Slicescope, Scientifica, UK). For the LSPS component (see Fig. 1), a dichroic mirror was used to direct light from a 375-nm laser diode (IQ2A, Power Technology, USA) towards a 4x microscope objective (0.1 NA), which focused 2-mW of light to a spot <10 *μ*m in diameter at the sample. The laser diode current was gated to generate a 2-ms pulse of light for each photostimulation event. When a light pulse illuminated a neuron, nearby caged glutamate was photolyzed within 0.2 *μ*s and caused the neuron to fire action potentials. Galvano-driven mirrors (not shown) under computer control were utilized to rapidly steer the laser spot to any point within a 1350- *μ*m×1350- *μ*m field-of-view (FOV) containing hundreds of neurons.

For the Ca imaging component, a different dichroic mirror was placed above the first and used to direct 494-nm light from a high-power light emitting diode (LED) (PE-100, CoolLED, UK) through the same microscope objective. This light broadly illuminated the entire FOV in order to excite Fluo-4 AM, a cell-permeant and calcium-sensitive dye. Because intracellular free-calcium (Ca ^2+^) increases whenever a neuron fires an action potential, large-scale activity of all neurons in the FOV can be recorded by detecting changes in the 516-nm emission fluorescence using a fast and sensitive EM-CCD camera (ImagEM, Hamamatsu, Japan).

Experiments were performed on primary cultures using rat cortical neurons dissociated from embryonic day 17 (E17) Sprague-Dawley rats and plated onto 12-mm glass coverslips pretreated with poly-L-ornithine. The plating medium consisted of 80% Dulbecco’s modified Eagle’s medium (DMEM) without glutamine, 10% Ham’s F12-nutrients, 10% bovine calf serum (heat-inactivated and Fe-supplemented), 25 mM HEPES stock, 24 U/ml penicillin, and 24 *μ*g/ml streptomycin. Cultures were seeded for one day at a density of ∼225,000 cells/ml. On day in vitro (DIV) 2, glial cell proliferation was inhibited with 150 *μ*M cytosine arabinoside and the medium was switched to growth medium consisting of neurobasal medium, 20 mM B-27 supplement, 24 U/ml penicillin, and 24 *μ*g/ml streptomycin. The growth medium was refreshed three times a week by replacing half of the volume. Final cell density was 300-400 cells/mm^2^, which contained both excitatory and inhibitory neurons.

Prior to each experiment, a coverslip was incubated for 30 min in growth medium containing 2 *μ*M Fluo-4 AM (Thermo Fisher Scientific, USA), a cell-permeant calcium indicator. The dye was transferred from stock solution by dissolving in equal volume Pluronic F-127 (20% in DMSO). The coverslip was then placed in imaging medium (pH 7.2) consisting of 5 mM KCl, 140 mM NaCl, 1 mM MgCl_2_, 1 mM CaCl_2_, 24 mM D-glucose, and 10 mM HEPES; and incubated for another 30 min. Finally, caged glutamate (MNI-glutamate, Tocris Bioscience, UK) was added to give a final concentration of 200 *μ*M. The 494-nm excitation light for Fluo-4 is sufficiently far away from 340-nm peak absorption wavelength for MNI-glutamate, to allow it to operate without interference. Indeed, auxiliary experiments verified that the presence of MNI-glutamate had no observable effect on the level of spontaneous activity.

Initially, cultured neurons were plated without making any connections. After DIV 3-4, neurons begin to form synaptic connections and randomly fire action potentials. At DIV 8-12, network bursting begins when about 20% of neurons fire together roughly every 2 min. As the network matured, both the intensity and frequency of bursting increased such that, at DIV 20, nearly 80% of neurons were firing together every 30 s.

## Mapping functional connectivity

**Fig. 2:**
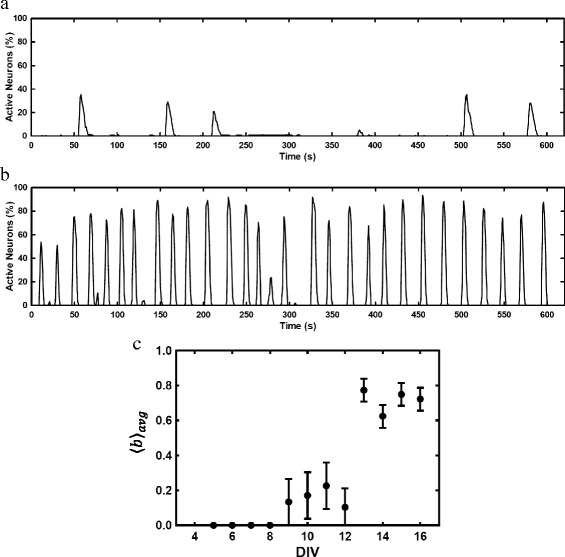
Neuronal network bursting. Percentage of spontaneously active neurons in mapped neuronal networks at **a** DIV 9 and **b** DIV 16. A neuron was considered active when it exhibited >1*%* Ca responses (*Δ* *F*/*F*). A network bursting event was defined to occur when ≥20*%* of mapped neurons were active within the same 1-sec time frame. The bursting parameter, **b**, characterized the fraction of the time a neuron participated in a network bursting event. **c** 〈*b*〉_*avg*_, which is the average of *b* over all mapped neurons and over all networks for that day, shows network bursting as a function of network age

A typical experiment began by recording spontaneous network activity for 10 min of neurons near the center of the coverslip. Although spiking activity typically occurs in short (∼ 10–50 ms) bursts of action potentials, the corresponding fractional changes in dye fluorescence, or Ca responses (*Δ* *F*/*F*), are much longer (∼10 sec) (Cossart et al. 2005). Thus, images taken once per second at a 50-ms exposure time were sufficient to capture all network bursting activity. A neuron was considered active if its fluorescence changed by more than 1%. A network bursting event was defined to occur when ≥20*%* of neurons in the final mapped network (to be determined later) were active within the same 1-sec time frame. To characterize network bursting for each neuron, we defined the bursting parameter, *b*, to be the fraction of time a neuron participated in a network bursting event. This parameter was then averaged over all mapped neurons, 〈*b*〉, and then over all networks for that day, 〈*b*〉_*avg*_ and indicates the level of network bursting activity as a function of network age. As is similarly reported in work by others (Tibau et al. 2013; Wagenaar et al. 2006), a clear transition to network bursting was observed at DIV 13 [Fig. 2 c].

**Fig. 3:**
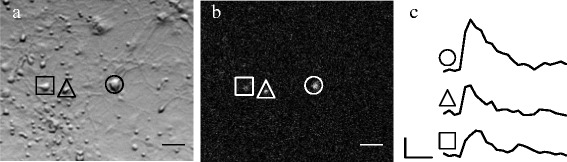
Simultaneous laser scanning photostimulation (LSPS) and calcium (Ca) imaging. **a** Bright-field and image showing positions of photostimulated neuron (*circle*) and responding neurons (*triangle* and *square*). Scale bar: 50 *μ*m. **b** Ca responses (fractional change in Fluo-4 fluorescence) 2 s after photostimulation. Scale bar: 50 *μ*m. **c** Ca responses of corresponding neurons as a function of time. Scale bars: 5% (*vertical*) and 5 s (*horizontal*)

Next, using the same camera frame-rate and exposure time, functional mapping began by first stimulating a randomly chosen neuron near the center of the FOV and identifying neurons that showed changes in fluorescence. Single-neuron photostimulation could be achieved because the laser spot size (<10 *μ*m) was comparable to the size of a neuron and much less than the typical interneuronal spacing in these cultures. Typically, responding neurons were ∼100 *μ*m from the photostimulated neuron and, thus, well within the FOV area. Because the Fluo-4 rise time (∼20 ms) is much less than the time between frames, Ca responses from neurons that were directly connected to the photostimulated neuron, i.e. first order neurons, began at the same imaging frame as the response from the photostimulated neuron [see Fig. 3]. While Ca responses from second order neurons could be elicited by increasing the light power and/or pulse duration, these had onset times of 3– 5 s after photostimulation and could easily be distinguished. The longer time delay is presumably due to postsynaptic integration rather than synaptic delay time (typically 2– 5 ms). Based on these observations, first order neurons were chosen along two criteria: 1) the Ca responses occurred on or after photostimulation and 2) had to be ≥50*%* of its peak value 2 s after photostimulation.

Mapping proceeded by individually photostimulating these first order neurons, then their neighbors, and so on. When the list of photostimulation targets was exhausted, another neuron was randomly selected and the mapping process continued again. Because Ca responses at this level of sensitivity correspond to supratheshold events (i.e. action potentials), only excitatory connections were mapped. Custom-written software (LabView, National Instruments, USA) allowed the mapping process to be almost fully automated – user intervention was only required to approve responses that satisfied the criteria. Because Fluo-4 is toxic to cells after ∼4 h, the mapping process ended after 2– 3 h and the coverslip was discarded. Within this time, raw map data consisting of ∼200 neurons and ∼2000 connections were typically made. (In principle, the “approval” process can be automated as well, and the number of cells that could be mapped will only limited by the 20 s it takes to record a complete Ca response). In one day, 1–3 networks of similar age, i.e. from same original batch, were usually mapped.

A number of exclusions were applied before obtaining the final map data. First, self-links were removed because, although autapses may exist in a neuronal culture, their associated Ca responses cannot be distinguished from the much larger direct photostimulation response. Moreover, self-links are usually not considered in most network analyses. Second, although it rarely occurred, nodes that had neither incoming nor outgoing connections were removed. Last, to consider only a completely mapped network, we removed outgoing links to nodes that were not photostimulated due to time constraints. These links represented about 10% of the total and had little effect on the overall network density, which remained sparse at about 5% for all networks.

## Results

**Fig. 4:**
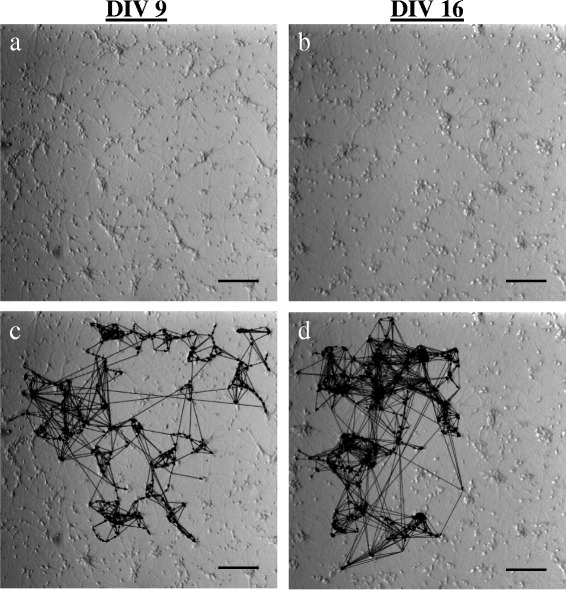
Connectivity maps. For the same two networks shown in Fig. 2, bright-field image of **a** small (DIV 9) and **b** large (DIV 16) network bursting activity. Corresponding connectivity maps overlaid onto bright-field images: **c** 195 nodes and 874 links; **d** 186 nodes and and 1567 links. Scale bars: 200 *μ*m

**Fig. 5:**
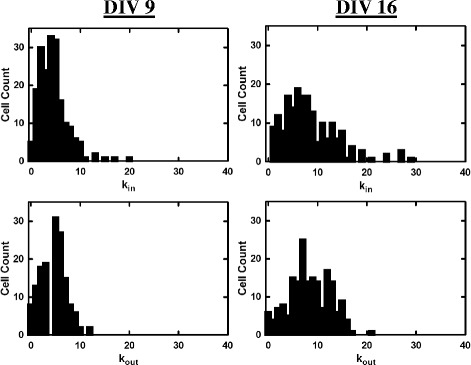
Degree distributions of the same two networks shown in Fig. 4

From this final map data, the adjacency matrix for an unweighted and directed network was constructed and used to calculate network properties. For each neuron, we first investigated the number of incoming connections, or in-degree (*k*_*in*_), and the number of outgoing connections, or out-degree (*k*_*out*_). Figure 4 shows examples of two networks at two different stages of network bursting activity (DIV 9 and DIV 16). In general, the corresponding degree distributions (Fig. 5) were single-peaked and did not display the power law dependence of scale-free networks (Barabási 1999).

**Fig. 6:**
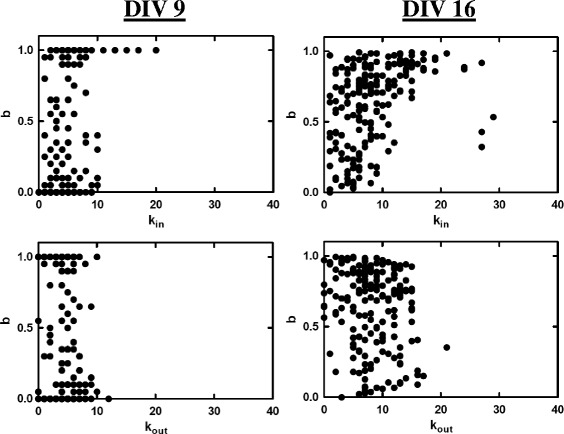
Bursting parameter vs. in-degree (out-degree) plotted for each neuron in the same two networks shown in Fig. 4

**Fig. 7:**
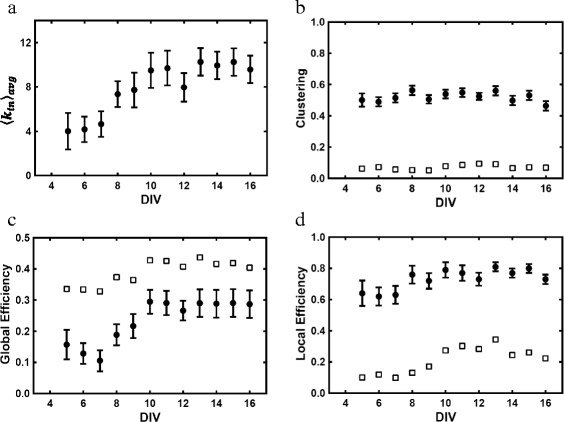
Network properties. **a** Average in-degree, **b** clustering, **c** global efficiency, and **d** local efficiency as a function of neuronal age. *Solid circles*: averages of all networks mapped for given day with error bars determined by the standard error of the mean (SEM). *Open squares*: averages computed from a randomized network model having the same degree distribution as each mapped network

Correlation between degree and bursting activity was explored [Fig. 6]. Correlation was only found for in-degree (Pearson’s correlation with *r*=0.3−0.5). As may be expected from simple models of neuronal integration, neurons with large *k*_*in*_ tended to burst more. However, the converse relationship was not true as there were many neurons with large bursting parameters but small *k*_*in*_. This suggests that either they possess relatively large input strengths or their intrinsic spontaneous activity was already well-synchronized with network bursting. The average in-degree for each network was found and then further averaged over all networks mapped on the same day to get 〈*k*_*in*_〉_*avg*_, which increased from initial values of 〈*k*_*in*_〉_*avg*_≃4 to a stable values of 〈*k*_*in*_〉_*avg*_≃10 after the onset of network bursting [Fig. 7 a]. Using higher density cultures and different methods, other investigations have reported values that are larger by a factor ∼5 (Soriano et al. 2008; Stetter et al. 2012).

We looked at clustering, which is the tendency for a network to form tightly connected neighborhoods. The clustering coefficient *C*_*i*_ (as defined for a directed network (Fagiolo 2007)) was calculated for each neuron. This parameter quantifies the likelihood that a node’s neighbors are themselves neighbors to each other. Values of *C*_*i*_ were averaged over all neurons and networks for a given day and compared to those calculated from a randomized network model that preserves degree distribution (Rubinov and Sporns 2010). We found that while clustering was much larger with 〈*C*〉_*avg*_≃0.5 than in the random network model [Fig. 7 b], it was relatively constant over the entire period, and thus uncorrelated with network bursting.

We calculated global efficiency, which measures how well information propagates over the network (Latora and Marchiori 2001) and is given by

1 $$ E_{G}=\frac{1}{N(N-1)}\sum_{i\neq j} \frac{1}{d_{ij}}  $$

where *N* is the number of nodes and *d*_*ij*_ is the shortest path (number of links) between any two nodes *i* and *j* in a directed network. Thus, the closer two nodes are to each other, the higher the global efficiency, while disconnected nodes make no contribution. Figure 7 c shows the average global efficiency for each day rapidly increasing from an initial value of ≃0.13 to a steady value of ≃0.29 within four days (DIV 7-10) before the onset of network bursting. By contrast, global efficiency of the random network model stays relatively high throughout.

The ‘small-world’ behavior of these networks was also assessed. To do this, we considered the local efficiency *E*_*L*_ of a node, which is defined similarly to Eq.1 but only applied to the node’s neighbors instead of the entire network (Latora and Marchiori 2001). It is related to the clustering coefficient and measures how well communication is relayed by the node’s neighbors if the node is removed. Using a modified algorithm found in the Brain Connectivity Toolbox (Rubinov and Sporns 2010), we found the local efficiency by averaging over all nodes and then networks for each day. The initial value was large 〈*E*_*L*_〉_*avg*_≃0.63 and increased by roughly 20% over time [Fig. 7 d]. As discussed in (Latora and Marchiori 2003), these networks exhibited ‘small-world’ network behavior because both *E*_*G*_ and *E*_*L*_ remained relatively large throughout the entire time period. As expected, the random network model did not show this behavior as its local efficiency remained relatively low.

## Discussion

We have described here a technique that allows single-cell photostimulation while simultaneously recording spiking activity of a relatively large population of neurons. To our knowledge, such a combination of methods has not been reported elsewhere. We used this technique to directly measure the functional connectivity of cultured neurons before and after the appearance of network bursting. Connectivity maps, each consisting of ∼200 neurons and ∼2000 connections, were made over a 12-day time period. Network properties determined from these data show that average node degree and global efficiency correlate with network bursting, while preserving ‘small-world’ behavior, and suggest that network structure may indeed play a role in initiating and synchronizing this type of neuronal oscillation exhibited by cultured neurons.
